# Non-disclosed men who have sex with men within local MSM HIV-1 genetic transmission networks in Guangyuan, China

**DOI:** 10.3389/fpubh.2022.956217

**Published:** 2022-08-09

**Authors:** Lacuo Zhuoma, Yan Zhang, Tu Yan, Fayang Kang, Xueqin Hou, Jianguo Chen, Min Huang, Yali Zeng, Qiushi Wang, Chang Zhou, Shu Liang, Ling Su

**Affiliations:** ^1^Center for AIDS/STD Control and Prevention, Sichuan Center for Disease Control and Prevention, Chengdu, China; ^2^Guangyuan Center for Disease Control and Prevention, Guangyuan, China; ^3^Lizhou District Center for Disease Control and Prevention, Guangyuan, China

**Keywords:** HIV, phylogenetic analysis, non-disclosed, MSM, factors

## Abstract

**Background:**

Most men who have sex with men (MSM), especially those with HIV infection, do not disclose their same-sex behaviors in China due to Chinese family values and fear of stigmatization, rejection, or prejudice. However, disclosure of same-sex behaviors to healthcare providers (HCPs) can be beneficial for reducing viral transmission and promoting their physical and mental health. In this study, by combining phylogenetic analysis with traditional epidemiological approaches, we tried to identify the MSM who do not disclose to HCPs in transmission networks and explored the factors related to the non-disclosed behaviors.

**Method:**

Phylogenetic analysis was conducted using HIV *pol* sequences obtained from the drug-resistant surveillance program, which was collected as part of routine clinical care since 2012. Sequences were linked to the demographic data collected in the Chinese HIV/AIDS Comprehensive Response Information Management System (CRIMS). First, male patients in whom genetic sequences were within the molecular transmission clusters involving self-reported MSM were identified as potential MSM (pMSM). Then, a cross-sectional survey was conducted to supplement behavioral information and attitudes toward MSM.

**Results:**

Our sample consisted of 190 pMSM patients. In total, 43.16% of the patients were likely to conceal same-sex behaviors during the first-self-report, and 14.73% of patients might continue to conceal a history of same-sex behaviors even after receiving medical care. The pMSM who concealed their same-sex behaviors were reluctant to accept medical services such as Voluntary Counseling and Testing (VCT) and had a lower likelihood of condom use. In addition, the related factors for non-disclosed behavior were associated with current address, income before diagnosis, and attitudes toward MSM.

**Conclusion:**

Non-disclosure of same-sex behaviors to HCPs may be a major obstacle for certain medical services for MSM who exhibit risky sexual behaviors. The pMSM from developing areas, with high monthly income, and with neutral or un-supportive attitudes toward MSM may represent non-disclosure of their same-sex behaviors. Thus, policies facilitating MSM to disclose their same-sex behaviors are recommended, such as legislations protecting homosexual rights on employment, education, marriage, and so on.

## Introduction

In most countries, the prevalence of HIV among men who have sex with men (MSM) is higher than in the general population and continues to rise (1). Since the late 1990s, a wealth of data have shown that MSM is significantly affected by HIV prevalence, and there is a rising trend in patients newly infected with HIV in China (2, 3). Results from the national HIV sentinel surveillance have found that the HIV infection rates of MSM in China increased from 5.73% in 2010 to 7.98% in 2015 (4). Even so, reported currently is that the number of MSM cases with HIV infection is still considered to be an underestimate in China (5).

In traditional Chinese culture, homosexuality is large disobedience of family values (6). MSM in China is less likely to disclose their same-sex behaviors to others compared with those in western countries (7), owing to HIV/AIDS-related behaviors and stigma, social capital and acculturation, and some demographic characteristics. The disclosure of same-sex behaviors by MSM is situational and context based and with different sets of factors, which are associated with their selective disclosure to different audiences (8). Notably, the disclosure rate of Chinese MSM to doctors or healthcare providers (HCPs) is significantly low (8). More than 80% of MSM have not disclosed their sexual orientation to health professionals (9). For MSM, reporting their same-sex behaviors to HCPs (disclosure to HCPs) may promote their linkage to HIV prevention and treatment cascade and improve their health outcomes (10). The previous data have shown that MSM without disclosing their same-sex behaviors are more likely to exhibit bisexual behavior and risky sexual behavior, for example, not using condoms (11). More worrisome is the non-disclosed MSM would miss receiving MSM-targeted health services, such as pre-exposure prophylaxis (PrEP), post-exposure prophylaxis (PEP), and HIV testing (5).

Phylogenetic analysis has been confirmed as an effective measure to identify and dissect HIV transmission clusters (12) and to infer potential transmission chains or networks between HIV infections (13, 14). An increasing number of studies support that molecular network analysis could contribute to revealing the transmission risk groups and hotspots and estimating the speed of HIV transmission (15). Combined with detailed epidemiological and clinical surveillance data, HIV molecular transmission networks provide a chance to identify potential non-disclosed MSM, and then, analyze the possible causes of the non-disclosure of same-sex behaviors.

## Materials and methods

### Study design and sampling methods

A phylogenetic analysis was performed that uses special sequences of HIV pol amplified from patient samples obtained from the drug-resistant surveillance program in Guangyuan city, which was collected as part of routine clinical care since 2012. A local HIV-1 genetic transmission network had been constructed, and we selected male patients whose genetic sequences were within the molecular transmission clusters involving self-reported MSM in the Chinese HIV/AIDS Comprehensive Response Information Management System (CRIMS). These samples of male patients older than 18 years with long-term medical care for more than 1 year in those clusters were defined as potential MSM (pMSM). A cross-sectional survey targeting “pMSM” was conducted from November to December 2021 in Guangyuan city of Sichuan province, China. The survey was conducted by HCPs engaged in the follow-ups of people living with HIV/AIDS through face-to-face or telephone interviews. Participants were verbally informed of the purposes and procedures of the study, the sensitive nature of the questions, the level of confidentiality, the compensation, and their rights to refuse or stop the interview. Next, study participants were asked to verbally express their understanding and consent of the study. This study was reviewed and approved by the ethics committee of the Sichuan Center for Disease Prevention and Control.

### Genetic analysis of HIV-1 transmission network

Sequences were generated from the HIV-1 pol. The Reverse Transcription-Polymerase Chain Reaction (RT-PCR) was used to amplify the full-length protease gene in the pol region and the first 300 codons of the reverse transcriptase gene (16). Local HIV-1 sequences were entered into the HyPhy software to calculate their genetic distances, and Tamura-Nei93 pairwise genetic distances were calculated for all pairs of sequences. Two sequences showing a genetic distance of ≤ 1.5% were identified as potential transmission partners (17). Finally, the network data were visualized and analyzed using the network software Cytoscape 3.5.1 (18).

### Data collection

We extracted part of the data from CRIMS (19), including basic demographic characteristics, such as age, education, marital status, occupation and current address, self-reported transmission route, time of confirmed HIV positivity, and the first CD4 results. Then, a self-designed questionnaire was used to collect information such as pre-diagnosis conditions, including time of local residence, monthly income, and the approach to HIV testing (VCT, PITC, and others) when tested positive, with/without heterosexual partners. In addition, participants were asked whether they had sex with same-sex partners, and their attitudes toward MSM, which was conducted using the Gay Attitude Scale compiled by Yu Yong (20). The scale used the Likert 5-level scoring method, with the higher score indicating a more supportive attitude.

For the participants who reported same-sex behaviors in the survey, the same-sex behavior model and same-sex dating model were further investigated, including the first same-sex sexual age, the average monthly numbers of same-sex partners before diagnosis, same-sex partner type before diagnosis, and condom use with the same-sex partner in the last month before diagnosis.

### Definitions

We classified pMSM into three categories based on self-reported sexual behavior in CRIMS and/or in the survey. (1) **Self-reported MSM**, who reported themselves as MSM in CRIMS, and also disclosed their same-sex behaviors in the survey. (2) **Medical care-reported MSM**, refers to those pMSM who first reported themselves as heterosexual in CRIMS, while under receiving long-term medical care, they finally disclosed their same-sex behaviors in the survey. (3) **Self-reported heterosexual man**, the others who self-reported as heterosexual in both CRIMS and the survey.

### Statistical analysis

All data analyses were completed using SPSS 23. For the descriptive analysis, we compared basic demographic characteristics and behavioral information obtained from the survey of the participants who had disclosed their same-sex behaviors to those who had not, and Chi-square test was performed to examine the relationships among variables of interest. Univariate and multivariate logistic regressions were used to evaluate factors associated with same-sex behaviors disclosure among the participants. A total of 15 variables were tested. Each of these variables was first tested using univariate modeling and these variables with a *p* < 0.05 at this step were considered eligible for the multivariate analysis. The significance level was fixed at α = 0.05.

## Results

### Socio-demographics and attitudes toward MSM

**Figure 1:**
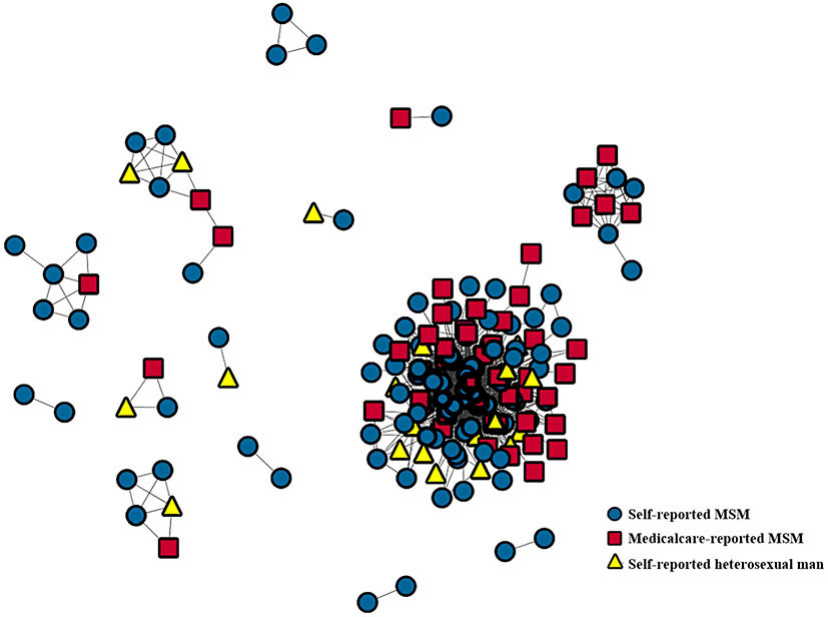
Positions of three types of pMSM within the sequences clusters. Sequences were generated from the HIV-1 pol. All the sequences contained in networks of pMSM were analyzed by constructing a local MSM HIV-1 genetic transmission network. Medical care-reported MSM and self-reported heterosexual men were always linked to self-reported MSM.

A total of 283 male patients were identified as pMSM, whose genetic sequences were within the self-reported MSM clusters. However, 93 pMSM were transferred to treatment elsewhere and could not be contacted, or refused to be investigated. Thus, our sample consisted of 190 pMSM patients. All the sequences contained in networks of pMSM were analyzed by constructing a local MSM HIV-1 genetic transmission network. As shown in Figure 1, 108(56.8%) patients were self-reported MSM. In total, 54 (28.4%) medical care-reported MSM first reported themselves as heterosexual in CRIMS, and then disclosed their same-sex behaviors in the survey. In total, 28 (14.7%) still self-reported as heterosexual men who never had same-sex behaviors.

Next, we also analyzed the potential correlations for the socio-demographic characteristics and attitudes toward MSM among the three classes of pMSM by using the Chi-square test. Our data as shown in Table 1 found that 54.7% (*n* = 104) of pMSM kept neutral attitude and 13.7% (*n* = 26) kept un-supportive attitude to MSM. In addition, the related factors led to different self-reported sexual behaviors among the pMSM. Those pMSM might be likely to self-report as MSM who had never married, living in central downtown, finding HIV positive by VCT approach, without heterosexual partners, whose first CD4 results ≥200 cells/μl, keeping supportive attitude to MSM (*p* < 0.05).

**Table 1:** Demographic characteristics and attitudes toward MSM among pMSM (%).

	**Overall (190)**	**Self-reported MSM (108)**	**Medical care-reported MSM (54)**	**Self-reported heterosexual man (28)**	**χ^2^**	***P***
**Age group, years**						
<40	107(56.3)	68(63.0)	25(46.3)	14(50.0)	4.60	0.10
≥40	83(43.7)	40(37.0)	29(53.7)	14(50.0)		
**Time of confirmed HIV positivity**						
Before 2015	62(32.6)	39(36.1)	13(24.1)	10(35.7)	6.26	0.18
2015–2018	89(46.8)	52(48.1)	24(44.4)	13(46.4)		
2019–2021	39(20.5)	17(15.7)	17(31.5)	5(17.9)		
**Education**						
Middle school or below	65(34.2)	29(26.9)	24(44.4)	12(42.9)	7.99	0.09
High school	86(45.3)	55(50.9)	18(33.3)	13(46.4)		
College degree or above	39(20.5)	24(22.2)	12(22.2)	3(10.7)		
**Marital status**						
Married	44(23.2)	14(13.0)	16(29.6)	14(50.0)	21.13	<0.01
Never married	101(53.2)	69(63.9)	23(42.6)	9(32.1)		
Divorced or Widowed	45(23.7)	25(23.1)	15(27.8)	5(17.9)		
**Occupation**						
Farmer	71(37.4)	29(26.9)	26(48.1)	16(57.1)	15.79	0.01
Student	13(6.8)	11(10.2)	2(3.7)	0		
Others	106(55.8)	68(63.0)	26(48.1)	12(42.9)		
**Current address**						
Surrounding county	69(36.3)	33(30.6)	10(18.5)	26(92.9)	54.75	<0.01
central downtown	105(55.3)	70(64.8)	35(64.8)	0		
suburb	16(8.4)	5(4.6)	9(16.7)	2(7.1)		
**Floating population or not**						
Yes	147(77.4)	78(72.2)	41(75.9)	28(100.0)	9.89	0.01
No	43(22.6)	30(27.8)	13(24.1)	0		
**AIDS reporting area**						
Guangyuan	153(80.5)	90(83.3)	44(81.5)	19(67.9)	3.44	0.18
Others	37(19.5)	18(16.7)	10(18.5)	9(32.1)		
**Time of local residence**						
≤ 3 years	28(14.7)	14(13)	12(22.2)	2(7.1)	3.96	0.14
>3years	162(85.3)	94(87)	42(77.8)	26(92.9)		
**Monthly income before diagnosis, RMB**						
<3,000	94(49.5)	53(49.1)	34(63.0)	7(25.0)	10.65	0.01
≥3,000	96(50.5)	55(50.9)	20(37.0)	21(75.0)		
**The approach to HIV testing when tested positive**						
VCT	68(35.8)	59(54.6)	8(14.8)	1(3.6)	48.64	<0.01
PITC	85(44.7)	32(29.6)	38(70.4)	15(53.6)		
Others	37(19.5)	17(15.7)	8(14.8)	12(42.9)		
**With/Without heterosexual partners**						
No	113(59.5)	77(71.3)	35(64.8)	1(3.6)	43.21	<0.01
Yes	77(40.5)	31(28.7)	19(35.2)	27(96.4)		
**The first CD4 results (cells/μl)**						
<200	47(24.7)	16(14.8)	14(25.9)	17(60.7)	25.22	<0.01
≥200	143(75.3)	92(85.2)	40(74.1)	11(39.3)		
**Attitudes toward MSM**						
Supportive	60(31.6)	43(39.8)	15(27.8)	2(7.1)	48.31	<0.01
Neutral	104(54.7)	58(53.7)	35(64.8)	11(39.3)		
Un-supportive	26(13.7)	7(6.5)	4(7.4)	15(53.6)		

*VCT, Voluntary Counseling and Testing; PITC, Provider Initiated HIV Testing and Counseling*.

### Same-sex behaviors and dating models

In total, 162 pMSM disclosed their same-sex behaviors in the survey. The mean age of the first same-sex behavior was 24.4 ± 7.5 years old. Our data as shown in Table 2 showed that age was associated with disclosure MSM, supported by the linear correlation coefficients that the younger the first same-sex sexual age, the higher the proportion of self-reported MSM (39.8% vs. 22.2%; *p* < 0.05). Expectedly, self-reported MSM were more likely to have regular partners before HIV diagnosis (48.1% vs. 25.9%; *p* < 0.05).

**Table 2:** Same-sex behaviors and dating models among pMSM self-reporting having happened same-sex behavior in the survey (%).

	**Overall (162)**	**Self-reported MSM (108)**	**Medical care-reported MSM (54)**	**χ^2^**	***P***
**The first same-sex sexual age**					
<20	55(34.0)	43(39.8)	12(22.2)	6.47^*^	0.01
20–29	71(43.8)	46(42.6)	25(46.3)		
≥30	36(22.2)	19(17.6)	17(31.5)		
**The average monthly numbers of same-sex partners before diagnosis**
1	45(27.8)	27(25.0)	18(33.3)	1.59	0.45
2	70(43.2)	47(43.5)	23(42.6)		
≥3	47(29.0)	34(31.5)	13(24.1)		
**Same-sex partner type before diagnosis**					
Mainly with regular partner	66(40.7)	52(48.1)	14(25.9)	12.81	<0.01
Mainly with temporary partner	92(56.8)	56(51.9)	36(66.7)		
Mainly with Commercial partner	4(2.5)	0	4(7.4)		
**Condom use with the same-sex partner in the last month before diagnosis**					
Frequently use	29(17.9)	25(23.1)	4(7.4)	7.17	0.03
Occasionally use	97(59.9)	58(53.7)	39(72.2)		
Never use	36(22.2)	25(23.1)	11(20.4)		
**Same-sex dating models**					
Online	92(56.8)	62(57.4)	30(55.6)	8.46	0.03
Offline	14(8.6)	10(9.3)	4(7.4)		
Both online and offline	47(29.0)	34(31.5)	13(24.1)		
Vacancy	9(5.6)	2(1.9)	7(13)		

* *Chi-squared test for linear trends*.

### Factors associated with disclosure after medical care

We analyzed the related factors for disclosure after medical care by comparing self-reported MSM (*n* = 108) with medical care-reported MSM (*n* = 54). Multivariate modeling demonstrated that compared to surrounding county, pMSM from suburbs were more likely to disclose their same-sex behaviors only after receiving medical care, with adjusted *ORs* (α*OR*) of 7.37 (95%*CI*:1.61–33.71). In addition, pMSM who reported HIV infection *via* Provider Initiated HIV Testing and Counseling (PITC) were more likely to disclose their same-sex behaviors only after receiving medical care, compared to Voluntary Counseling and Testing (VCT) (α*OR* = 10.51, 95%*CI*:3.92–28.18). Compared to self-reported MSM, the likelihood of condom use among medical care-reported MSM was lower (α*OR* = 5.23, 95%*CI*:1.44–19.01) (Table 3).

**Table 3:** Factors correlated with disclosure of same-sex behaviors after medical care.

	**Univariate analysis**				**Multivariate analysis**			
	**β**	***P***	***OR***	**95%*CI***	**β**	***P***	***αOR***	**95%*CI***
**Current address**								
Surrounding county			1.00				1.00	
Central downtown	0.50	0.23	1.65	0.73–3.73	0.96	0.06	2.61	0.95–7.16
Suburb	1.78	0.01	5.94	1.62–21.84	2.00	0.01	7.37	1.61–33.71
**Occupation**								
Farmer			1.00				1.00	
Student	−1.60	0.05	0.20	0.41–1.00	−1.37	0.14	0.25	0.04–1.58
Others	−0.85	0.02	0.43	0.21–0.86	−1.35	0.01	0.26	0.1–0.67
**Marital status**								
Married			1.00					
Never married	−1.23	0.00	0.29	0.12–0.68				
Divorced or Widowed	−0.64	0.19	0.53	0.20–1.37				
**The approach to HIV testing when tested positive**								
VCT			1.00				1.00	
PITC	2.17	<0.01	8.76	3.64–21.02	2.35	<0.01	10.51	3.92–28.18
Others	1.24	0.03	3.47	1.13–10.62	1.49	0.02	4.44	1.25–15.80
**The first same-sex sexual age**								
<20			1.00					
20–29	0.67	0.10	1.95	0.87–4.35				
≥30	1.17	0.01	3.21	1.28–8.01				
**Same-sex partner type before diagnosis**								
Mainly with regular partner			1.00					
Mainly with temporary partner	0.87	0.02	2.39	1.16–4.92				
Mainly with Commercial partner	22.52	1.00	–	–				
**Condom use with the same-sex partner in the last month before diagnosis**								
Frequently use			1.00				1.00	
Occasionally use	1.44	0.01	4.20	1.36–13.02	1.65	0.01	5.23	1.44–19.01
Never use	1.01	0.12	2.75	0.77–9.80	0.03	0.97	1.03	0.23–4.62

*VCT, Voluntary Counseling and Testing; PITC, Provider Initiated HIV Testing and Counseling*.

### Factors associated with deep non-disclosure of same-sex behaviors

Medical care-reported MSM (*n* = 54) and self-reported heterosexual men (*n* = 28) were analyzed to explore the factors associated with deep non-disclosure of same-sex behaviors. Multivariate modeling data demonstrated that compared to surrounding counties, pMSM from suburbs had a high probability to disclose their same-sex behaviors after medical care, with adjusted *ORs* (α*OR*) of 0.04 (95%*CI*:0–0.42). However, these pMSM with a monthly income ≥3,000 RMB before HIV diagnosis usually concealed their same-sex behaviors even under medical care (α*OR* = 20.33, 95%*CI*:2.06–200.98). Furthermore, in contrast to supportive attitudes, pMSM with neutral or un-supportive attitudes probably concealed their same-sex behaviors even if receiving medical care (α*OR* = 47.78, 95%*CI*:3.07–742.62) (Table 4).

**Table 4:** Factors correlated with non-disclosure of same-sex behaviors even after medical care.

	**Univariate analysis**				**Multivariate analysis**			
	**β**	***P***	***OR***	**95%*CI***	**β**	***P***	***αOR***	**95%*CI***
**The first CD4 results (cells/μl)**								
<200			1.00					
≥200	−1.49	<0.01	0.23	0.09–0.60				
**Current address**								
Surrounding county			1.00				1.00	
central downtown	−22.16	0.99	0.00	0.00	−22.59	1.00	0.00	0.00
suburb	−2.46	<0.01	0.09	0.02–0.47	−3.28	0.01	0.04	0–0.42
**Floating population or not**								
Yes			1.00				1.00	
No	−20.82	1.00	0.00	0.00	−21.51	1.00	0.00	0.00
**Monthly income before diagnosis, RMB**								
<3,000			1.00				1.00	
≥3,000	1.63	<0.01	5.10	1.84–14.12	3.01	0.01	20.33	2.06–200.98
**The approach to HIV testing when tested positive**								
VCT			1.00					
PITC	1.15	0.30	3.16	0.36–27.47				
Others	2.48	0.03	12.00	1.25–115.36				
**Attitudes toward MSM**								
Supportive			1.00				1.00	
Neutral/Un-supportive	1.61	0.04	5.00	1.05–23.71	3.87	0.01	47.78	3.07–742.62

*VCT, Voluntary Counseling and Testing; PITC, Provider Initiated HIV Testing and Counseling*.

## Discussion

This investigation consisted of 190 pMSM samples, and we observed that 43.2% of pMSM surveyed had not disclosed their same-sex behaviors to HCPs during the first-self-report, 28.4% probably disclosed their sexual behaviors or history of risky behaviors to HCPs after receiving medical care, and 14.7% had not disclosed their situation even if receiving medical care. There might be a fraction of heterosexual men who entered the MSM transmission cluster because of the female partners who had sex with bisexual MSM, such as a heterosexual wife of MSM (21). But it was still possible for those self-reported heterosexual men to deeply conceal their same-sex behaviors (22). As for the percentage of heterosexual men who were not concealed, further research is needed. Previous reports showed the higher rates of disclosure of same-sex behaviors in high-income countries, in part because of better social acceptance of homosexual relations. Several studies in the United States found that 60–90% of MSM had disclosed their same-sex behaviors to HCPs, and gay men were associated with higher odds of disclosure when compared to bisexual men (23–25). Non-disclosure of same-sex behaviors was a major barrier to providing clinical prevention and optimal care, and preventing transmission of HIV, thus, providers must be aware of patients' sexual orientation and behaviors (26, 27).

Similar to other domestic studies (5, 28), our correlation analysis showed that self-reported MSM represented the lowest proportion of heterosexual partners in this study. This indicated the non-disclosed MSM tended to have female sexual partners, and their bisexual behaviors led to a higher risk of HIV infection for females. Besides, the younger the first same-sex sexual age, the higher the proportion of self-reported MSM. Other domestic studies had found that the age of first same-sex behavior was usually before 20 years among positive self-identified gay (29). This might suggest that the earlier the MSM showed homosexual characteristics, the more likely they disclosed their same-sex behaviors to others.

By analyzing the potential factors for disclosure after medical care, we found that pMSM from suburb areas were more likely to disclose their same-sex behaviors only after receiving medical care. An investigation reported that MSM living in urban settings with higher education levels and higher income were more likely to disclose their sexual orientation to others (10). People living in suburbs generally had higher economic conditions and higher education levels than that in the surrounding counties but lagged behind central downtown. Thus, pMSM from suburbs might need to be driven by some conditions such as medical care before disclosing their same-sex behaviors. In addition, our study showed that in contrast to VCT, pMSM being identified with HIV infection *via* PITC were more likely to disclose their same-sex behaviors only after medical care. This indicated that the disclosed MSM were more likely to receive VCT service, suggesting the importance of promoting disclosure of same-sex behaviors in medical services. A study confirmed that unprotected anal intercourse was more prevalent among non-disclosed MSM (28). Similarly, we found that the likelihood of condom use among medical care-reported MSM was lower than self-reported MSM.

The analysis of pMSM with deep non-disclosure showed that monthly income (≥3,000 RMB) was associated with disclosure of their same-sex behaviors even after medical care, being different from another study (24). pMSM with supportive attitudes toward MSM were more likely to disclose their same-sex behaviors to others. This might be related to their comprehensive understanding of MSM and sexual knowledge.

Our study has several limitations. First, as some pMSM did not complete our survey and the data of the non-completers was excluded from our analysis, there might have been a selection bias as non-completers and participants might have had different socio-demographic characteristics and behaviors. And it also led to a decrease in the sample size of the study. Second, in the survey, we needed to ask about some pre-diagnostic behaviors, but the time of confirmed HIV positivity of every pMSM was different and it might lead to some recall bias because the length of recall varied across participants. Finally, as most collected data, especially the behavioral information, were self-reported, reporting bias might be present.

## Conclusions

Our study found that 43.2% of pMSM concealed same-sex behaviors to HCPs during the first-self-report and 14.7% continued to non-disclose after receiving medical care. In addition, our study demonstrated that same-sex behavior disclosure to HCPs was correlated with current address, income before diagnosis, and high-risk sexual behaviors and attitudes toward MSM. Non-disclosure of same-sex behaviors to HCPs also led to low acceptance of certain medical services. For example, non-disclosed MSM were less likely to receive VCT service. Thus, policies facilitating MSM to disclose their same-sex behaviors are recommended, such as legislations protecting homosexual rights on employment, education, marriage, and so on.

## Data availability statement

The original contributions presented in the study are included in the article/supplementary material, further inquiries can be directed to the corresponding author.

## Ethics statement

This study was reviewed and approved by the Ethics Committees of the Sichuan Center for Disease Prevention and Control under recording number SCCDCIRB2022-008. All participants were asked to verbally express their understanding and consent to the study. All methods were performed in accordance with the relevant guidelines and regulations.

## Author contributions

LZ, LS, and JC conceived and designed the study. LZ, YZh, TY, FK, XH, and MH were involved in the data collection. LZ and YZh analyzed the data and wrote the initial draft. YZe, QW, CZ, and SL provided critical comments. LS and QW provided constructive comments and revised the manuscript before submission. All authors contributed to the article and approved the submitted version.

## Funding

This study was supported by the Sichuan Province Science and Technology Support Program (2017JY0302). The funders had no role in study design, data collection and analysis, decision to publish, or preparation of the manuscript.

## Conflict of interest

The authors declare that the research was conducted in the absence of any commercial or financial relationships that could be construed as a potential conflict of interest.

## Publisher's note

All claims expressed in this article are solely those of the authors and do not necessarily represent those of their affiliated organizations, or those of the publisher, the editors and the reviewers. Any product that may be evaluated in this article, or claim that may be made by its manufacturer, is not guaranteed or endorsed by the publisher.
